# Constructing FeS and ZnS Heterojunction on N,S-Codoped Carbon as Robust Electrocatalyst toward Oxygen Reduction Reaction

**DOI:** 10.3390/nano13192682

**Published:** 2023-09-30

**Authors:** Fenglai Pei, Min Li, Yifan Huang, Qiuyun Guo, Kunming Song, Fantao Kong, Xiangzhi Cui

**Affiliations:** 1 Shanghai Motor Vehicle Inspection Certification & Tech Innovation Center Co., Ltd., Jiading District, Shanghai 201805, China; fenglaip@smvic.com.cn; 2 Shanghai Institute of Ceramics, Chinese Academy of Sciences, Shanghai 200050, China; limin232@mails.ucas.ac.cn (M.L.); huangyifan231@mails.ucas.ac.cn (Y.H.); 3 School of Chemistry and Materials Science, Hangzhou Institute for Advanced Study, University of Chinese Academy of Sciences, Hangzhou 310024, China; guoqiuyun22@mails.ucas.ac.cn (Q.G.); songkunming22@mails.ucas.ac.cn (K.S.)

**Keywords:** N,S codoped, ZnS/FeS, heterostructure, electron transfer, oxygen reduction reaction

## Abstract

Highly active and cost-efficient electrocatalysts for oxygen reduction reaction (ORR) are significant for developing renewable energy conversion devices. Herein, a nanocomposite Fe/ZnS-SNC electrocatalyst with an FeS and ZnS heterojunction on N,S-codoped carbon has been fabricated via a facile one-step sulfonating of the pre-designed Zn- and Fe-organic frameworks. Benefitting from the electron transfer from FeS to adjacent ZnS at the heterointerfaces, the optimized Fe/ZnS-SNC900 catalyst exhibits excellent ORR performances, featuring the half-wave potentials of 0.94 V and 0.81 V in alkaline and acidic media, respectively, which is competitive with the commercial 20 wt.% Pt/C (0.87 and 0.76 V). The flexible Zn-air battery equipping Fe/ZnS-SNC900 affords a higher open-circuit voltage (1.45 V) and power density of 30.2 mW cm^−2^. Fuel cells assembled with Fe/ZnS-SNC900 as cathodic catalysts deliver a higher power output of 388.3 and 242.8 mW cm^−2^ in H_2_-O_2_ and -air conditions. This work proposes advanced heterostructured ORR electrocatalysts that effectively promote renewable energy conversions.

## 1. Introduction

Oxygen reduction reaction (ORR) is an important electrochemical reaction process in clean and renewable energy conversion and storage technologies, e.g., fuel cells and metal–air batteries, whose output power densities are even determined by the ORR performance [1,2]. Platinum (Pt)-based catalysts are active toward ORR; unfortunately, however, they are considerably hindered in industrial applications because of their scarcity and high price [3]. Therefore, it is essential to develop low-cost and efficient non-precious ORR catalysts that overcome sluggish reaction kinetics and high overpotentials.

Metal-free doped carbons have attracted significant attention as efficient electrocatalysts for the adsorption of reactive intermediates during ORR, which can effectively regulate the electron and/or spin redistribution of the carbon matrix, thereby facilitating the ORR process [4,5,6]. It is well confirmed that S-doping is beneficial for the production of electron-withdrawing/donating groups, such as SOx-C or/and C-S-C species on the carbon plane [7], thereby modulating the electronic state of adjacent carbon atoms [8]. Moreover, the introduction of extra active units into heteroatoms-doped carbon substrates, including metal oxides, phosphides, sulfides, etc., is favorable for the further improvement of catalytic performance owing to co-catalysis [9,10,11,12]. Nowadays, the most widely investigated ORR electrocatalysts are mainly focused on Fe and Co metallic compounds [13,14]. In contrast, Zn-based materials have been very attractive for ORR due to their inexpensive and environmentally friendly properties.

Zeolitic imidazolate framework-8 (ZIF-8) has been widely employed as a precursor to fabricate the heteroatoms-doped porous carbons for high-efficiency electrocatalysts, such as Zn/N-codoped carbons [15,16,17]. Owing to their hierarchically porous morphology and modifiable elemental composition, ZIF-8 derivatives are proving to be promising alternatives to Pt-based ORR catalysts [18]. In particular, the Zn in the ZIF-8 can reconstructed with S atoms and connect with pyridinic-N to form a fast transport pathway for the adsorbed O-containing intermediates during ORR [19]. Moreover, the catalytic activity can be further improved by designing heterogeneous catalysts to tune the interface electrons [20,21,22]. These catalysts feature strong electronic coupling effects between different components, which is favorable for accelerating the electron transfer of interfaces [23]. For example, the Fe/Fe_3_C and FeS composite nanostructures have been proven to be highly efficient ORR electrocatalysts. Among them, FeS can accelerate the adsorptions of reaction substrates, but it delivers the sluggish kinetics of ORR. Fe/Fe_3_C could promote a 4e^-^-transferred ORR process but it has insufficient adsorption toward oxygen species. Yet only the heterostructures of Fe/Fe_3_C and FeS coupling could achieve a good catalytic effect of co-catalysis [24]. Therefore, the rational construction of heterostructured nanomaterials with hierarchical pores as advanced catalysts should be an effective strategy to improve ORR performance.

Herein, a nanocomposite Fe/ZnS-SNC catalyst with an FeS and ZnS heterojunction on N,S-codoped carbon has been fabricated via a facile one-step sulfonation treatment of the pre-designed Zn-/Fe-organic frameworks. The heterointerface between ZnS and FeS phases affords the formation of synergistic catalytic active centers, enabling the efficient charge transfer from FeS to ZnS sites at the interfaces to enhance the ORR performance of as-prepared catalysts. As a result, the resultant Fe/ZnS-SNC900 catalyst displays outstanding ORR activities with a half-wave potential of 0.94 V in alkaline and 0.81 V in acidic electrolytes, which are superior to the state-of-the-art Pt/C and most of the analogous catalysts. Attractively, the assembled flexible zinc–air battery using Fe/ZnS-SNC900 as air cathode exhibits higher open-circuit potential (1.45 V) and power density (30.2 mW cm^−2^), and the maximum power output of the Fe/ZnS-SNC900-equipped H_2_-O_2_ fuel cell reaches 388.3 mW cm^−2^. The research findings will facilitate the application of heterostructural metallic sulfides as advanced ORR catalysts in practical energy conversion systems.

## 2. Materials and Methods

### 2.1. Chemicals

Iron(III) chloride hexahydrate (FeCl_3_·6H_2_O, 99.0%), methanol (CH_3_OH, AR), and cetyltrimethylammonium bromide (C_19_H_42_BrN, 99%) were purchased from Sigma-Aldrich Chemical Reagent Co., Ltd. Zinc nitrate hexahydrate (Zn(NO_3_)_2_·6H_2_O, 99.0%), 2-methylimidazole (C_4_H_6_N_2_, 98.0%), and 2-aminobenzene-1,4-dicarboxylic acid (NH_2_-BDC, 98.0%) were obtained from Adamas Reagent Co. Ltd. Nafion D-520 dispersion (5 wt.%) was purchased from Dupont China holding. Commercial 20 wt.% and 40 wt.% Pt/C was purchased from Shanghai Hesen Electric Co., LTD. All chemicals were used without further purification.

### 2.2. Material Synthesis

Synthesis of Fe-NH_2_-BDC: First, 150 mg of FeCl_3_·6H_2_O and 50 mg of NH_2_-BDC were ultrasonically dispersed in 30 mL of deionized water, followed by stirring for 2 h. Then, the solution was poured into a Teflon-lined hydrothermal autoclave reactor and kept at 120 °C for 18 h. Finally, the resulting suspension was collected via centrifugation and washed with deionized water and methanol several times. The obtained solid powders (denoted as Fe-NH_2_-BDC) were dried at 60 °C under vacuum for 10 h.

Synthesis of ZIF-8: First, 1.2 g of Zn(NO_3_)_2_·6H_2_O was dissolved in 60 mL of deionized water, and then 5 mL of 0.01 M cetyltrimethylammonium bromide (CTAB) aqueous solution was injected and sonicated for 30 min. Then, the above solution was added to 100 mL of deionized water containing 16.0 g of 2-methylimidazole. The mixture solution was then ultrasonically treated for 10 min, followed by vigorous stirring at 1500 rpm for 3 h. The obtained precipitate was centrifuged and washed with deionized water and methanol several times and then freeze-dried overnight to obtain ZIF-8.

Synthesis of Fe-NH_2_-BDC@ZIF-8: The nanocomposite was synthesized using similar methods. First, 10 mg of Fe-NH_2_-BDC and 1.2 g of Zn(NO_3_)_2_·6H_2_O were dissolved in 80 mL of deionized water, and then 5 mL of 0.01 M CTAB aqueous solution was injected and sonicated for 30 min. Then, the above solution was added to 100 mL of deionized water containing 16.0 g of 2-methylimidazole. The mixture solution was then ultrasonically treated for 10 min, followed by vigorous stirring at 1500 rpm for 3 h. The resulting solid product (denoted as Fe-NH_2_-BDC@ZIF-8) was obtained by freeze-drying overnight. As a comparison, Fe-NH_2_-BDC-1@ZIF-8 was prepared through the same procedure apart from the input of Fe-NH_2_-BDC, which was increased to 50 mg.

Synthesis of Fe/ZnS-SNC: The obtained Fe-NH_2_-BDC@ZIF-8 was sulfurated in a tube furnace with two separate parts of 10 cm (S at the upstream) and then heated to 900 °C at 5 °C min^−1^ in Ar atmosphere. After the furnace was naturally cooled down, the sample was then obtained and named Fe/ZnS-SNC. For comparison, the Fe-NH_2_-BDC-1@ZIF-8, Fe-NH_2_-BDC, and ZIF-8 were also pyrolyzed via a similar process and named Zn/FeS-SNC, FeS-SNC, and ZnS-SNC, respectively. As a control, NC was also prepared by directly annealing ZIF-8 using the same procedure without S power. Zn/FeS-SNCX was also obtained via a similar preparation procedure except for the heat treatment temperature (X: 800, 900, 1000 °C).

### 2.3. Characterizations

X-ray diffraction (PXRD) patterns were collected from a Bruker D8 Advance diffractometer equipped with a Cu-Kα radiation source (40 kV, 40 mA). The surface morphology and elemental composition of samples were analyzed using a field-emission scanning electron microscopy (FE-SEM, Magellan 400) with an acceleration voltage of 5–30 kV. Transmission electron microscopy (TEM) images were performed on a field-emission transmission electron microscope (FETEM, JEM-F2100F) with a field-emission transmission electron microscope (200 kV). The X-ray photoelectron spectroscopy (XPS) was carried out using the Thermo Fisher Scientific ECSAlab250 XPS spectrometer with an Al-KαX-ray source (Thermo, Waltham, MA, USA). Raman spectra were examined on a Microscopic Confocal Raman Spectrometer (GX-PT-1500) with a 532 nm laser source.

### 2.4. Zinc–Air Batteries (ZABs)

The flexible Zn-air battery was assembled using a zinc foil as an anode, hydrophobic carbon paper coated with catalyst as an air cathode, and the gel polymer consisting of polyvinyl alcohol and KOH as a solid-state electrolyte. The zinc foil and catalyst-coated carbon cloth were placed on both sides of the gel film. The catalyst loading in the flexible Zn-air battery was 1.0 mg cm^−2^. For comparison, the battery using Pt/C (20 wt.%) as an air cathode was also equipped and evaluated under the same conditions. The polarization curve of zinc–air batteries was obtained on the CH 760E electrochemical workstation. All tests were carried out under ambient conditions.

### 2.5. Proton Exchange Membrane Fuel Cells (PEMFCs)

The performance of PEMFC was measured in the membrane electrode assembly (MEA) testing system (Model 850e, Scribner Associates, Southern Pines, NC, USA). The catalyst (15 mg) was ultrasonically dispersed in isopropanol (800 μL). Then, 80 μL of Nafion solution (5 wt.%) was added, and ultrasound continued for 30 min. The homogeneous catalyst ink was sprayed to one side of the membrane (Nafion-212, Dupont, Wilmington, DE, USA) as cathode, and the other side was modified with Pt/C (JM, 40 wt.%) ink as anode. The Pt loading was 0.1 mg cm^−2^ at the anode, and the loading of the as-prepared catalyst was confirmed to be 0.6 mg cm^−2^. The active area of the membrane electrode assembly (MEA) was 4 cm^2^. The fuel cell tests were carried out under H_2_-O_2_/air conditions with a gas flow rate of 200 sccm for H_2_ and 300 sccm for O_2_/air, and the back pressure was 200 KPa (relative humidity: 100%, cell temperature: 80 °C).

The electrochemical test methods and parameters are detailed in the Supplementary Materials.

## 3. Results

### 3.1. Synthesis and Characterization

**Figure 1:**
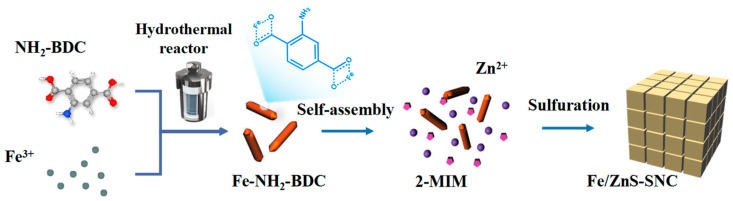
Schematic diagram for the preparation of Fe/ZnS-SNC.

As illustrated in Figure 1, the Fe/ZnS-SNC catalysts were fabricated through facile coordination self-assembly and subsequent sulfonation process via vapor deposition. Firstly, Fe-NH_2_-BDC was synthesized through a facile hydrothermal method; Fe(III) ions were coordinated with the carboxylate groups. After that, Fe-NH_2_-BDC and ZIF-8 composites were self-assembled via co-precipitation of Fe^3+^/Zn^2+^ and 2-methylimidazole (2-MIM). Finally, the FeS and ZnS nanoparticles were embedded into the N,S-codoped carbon via the sulfonation conversion of pre-designed Fe-NH_2_-BDC@ZIF-8 composites. The synthesis strategy is conducive to the formation of co-catalytic sites among polymetallic compounds.

**Figure 2:**
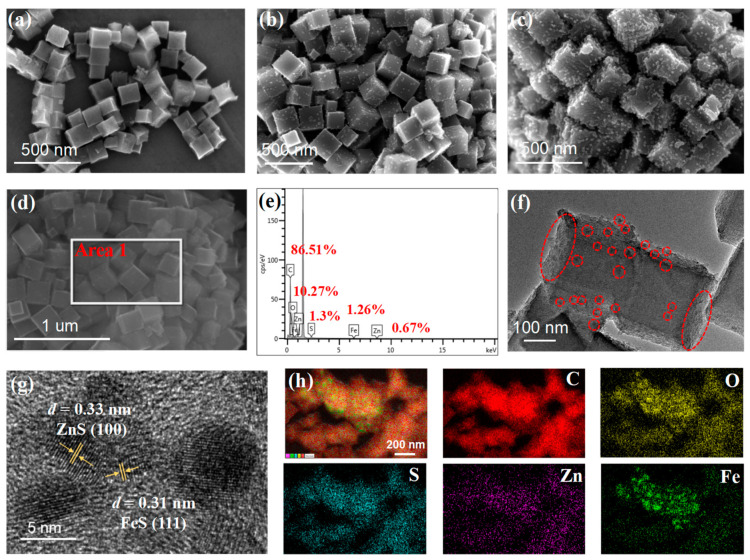
SEM images of NC (**a**), ZnS-SNC (**b**) and Fe/ZnS-SNC (**c**). SEM image of Fe/ZnS-SNC (**d**) with the corresponding SEM-EDS spectra (**e**) of area 1. TEM (**f**) and HR-TEM (**g**) images of Fe/ZnS-SNC. The element mapping of carbon, oxygen, sulfur, zinc, and iron in Fe/ZnS-SNC (**h**).

The morphologies of the NC, ZnS-SNC and Fe/ZnS-SNC are shown as SEM images in Figure 2a–c. Figure 2a exhibits the cubic morphology of NC, which is similar to that of ZIF-8 (Figure S1), indicating the structure of ZIF-8 has not been broken during pyrolytic process. After the sulfuration treatment (Figure 2b), the small ZnS nanoparticles are directly attached to the surface of NC. Fe/ZnS-SNC in Figure 2c shows a similar cubic morphology to ZnS-SNC, except for more nanoparticles observed on the surface, which is attributed to the coexistence of ZnS and FeS species. The SEM image of Zn/FeS-SNC (Figure S2) shows obvious metal agglomeration, suggesting that the introduction of excessive Fe species is not conducive to the formation of small metal sulfides. An energy-dispersive spectrometer (EDS) was used to analyze the elemental composition of the prepared Fe/ZnS-SNC sample in area 1 of Figure 2d. The EDS analysis confirms the existence of the carbon, oxygen, sulfur, iron, and zinc elements in the synthesized Fe/ZnS-SNC (Figure 2e). In addition, the EDS data also indicate the relative proportion of the different elements (inset in Figure 2e). Transmission electron microscopy (TEM) was carried out to analyze the microstructure of Fe/ZnS-SNC. As shown in Figure 2f, the nanoparticles marked with a red circle are clearly observed on the surface of the cubical structure, which is in accordance with the SEM image (Figure 2c). Furthermore, the high-resolution TEM (HR-TEM) shows the lattice spacing of nanoparticles at 0.31 and 0.33 nm, corresponding to the FeS (111) and ZnS (100) lattice planes [25], respectively (Figure 2g). These results indicate that the FeS and ZnS phases were constructed on N,S-codoped carbon during the high-temperature sulfuration, proving the formation of an FeS/ZnS heterojunction in Fe/ZnS-SNC. The EDS mapping (Figure 2h) of Fe/ZnS-SNC further verified the uniform distribution of C, O, S, Zn, and Fe elements on the catalyst surface.

**Figure 3:**
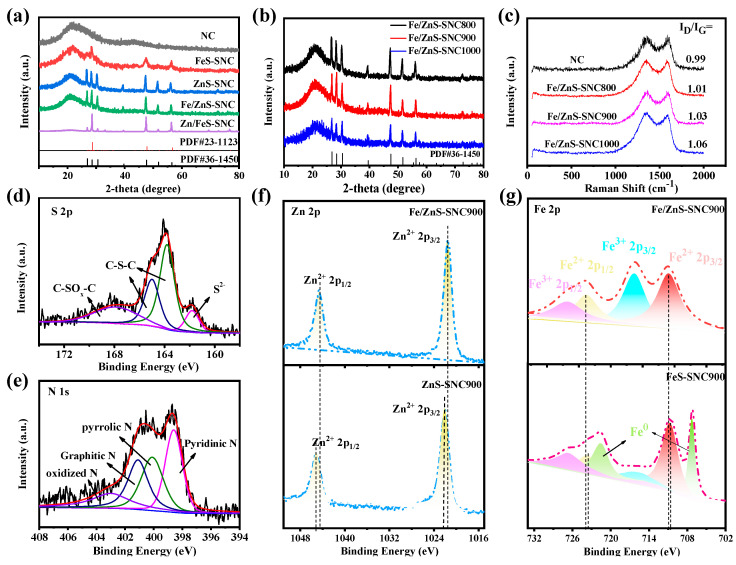
XRD patterns of different samples (**a**) and Fe/ZnS-SNCX (X: heat treatment temperature) (**b**). Raman spectra of NC and Fe/ZnS-SNCX (**c**). High resolution of S 2p (**d**) and N 1s XPS spectra (**e**) of Fe/ZnS-SNC900. The comparison diagrams of Zn 2p XPS spectra (**f**) in Fe/ZnS-SNC900 and ZnS-SNC900 and Fe 2p XPS spectra (**g**) in Fe/ZnS-SNC900 and FeS-SNC900.

The XRD pattern of prepared samples in Figure 3a exhibits a broad diffraction peak at about 2θ = 24°, which matches with the (002) plane of the graphite carbon and reveals a high graphitic degree [26]. The remaining peaks are assigned to ZnS (PDF#23-1123) and FeS (PDF#36-1450) phases, indicating the formation of ZnS and FeS composites after high-temperature sulfonation [27]. It is worth noting that with the increase in the Fe feed ratio, the proportion of the FeS phase increased significantly. In addition, the XRD patterns of the Fe/ZnS-SNC with different sulfuration temperatures are depicted in Figure 3b. The results show that the concentration of FeS decreased with the increase in sulfonation temperature. The Raman spectra in Figure 3c show two peaks at 1336 cm^−1^ and 1584 cm^−1^, corresponding to the D and G bands, which represent the amorphous and graphite carbon, respectively [28]. The intensity ratio of D to G band (I_D_/I_G_) is calculated to evaluate the disorder degree in sp^2^-hybrid carbon materials. The higher I_D_/I_G_ ratios imply the formation of more defects in the carbons, which is favorable for boosting the electrocatalytic performance of ORR. The intensity ratios of the D and G bands (I_D_/I_G_) were calculated as 0.99, 1.01, 1.03, and 1.06 for NC, Fe/ZnS-SNC800, Fe/ZnS-SNC900, and Fe/ZnS-SNC1000, indicating that more defects were produced with the formation of FeS/ZnS species and increased sulfuration temperature, which is the response for the elevated ORR activity in as-synthesized samples. The elemental composition and chemical state of the Fe/ZnS-SNC900 were analyzed using XPS measurements [29]. The XPS spectrum (Figure S3) reveals the existence of C, O, N, Zn, and Fe elements over the Fe/ZnS-SNC900 sample. The high-resolution S 2p spectrum (Figure 3d) of Fe/ZnS-SNC900 could be fitted into four peaks assigning for the metal sulfides (161.6 eV), thiophene sulfur (162.7 and 165.3 eV), and C-SOx-C (167.5 eV) [30], revealing the doping of S element via the sulfuration process and the formation of metal sulfides. The N 1s spectrum (Figure 3e) can be divided into four peaks at 403.2 eV (oxidized N), 401.1 eV (graphitic N), 400.0 eV (pyrrolic N), and 398.3 eV (pyridinic N), respectively [31]. The pyridinic N configurations have been proven to facilitate oxygen adsorption by providing more Lewis base sites in the neighboring carbon atoms, resultantly improving the higher intrinsic activity for the ORR. The peaks located at 1022.1 eV and 1044.5 eV can be observed in the Zn 2p_3/2_ and Zn 2p_1/2_ spectra, which is assigned to the Zn^2+^ (Figure 3f), confirming the presence of ZnS in Fe/ZnS-SNC900 [32]. The Fe 2p spectrum of Fe/ZnS-NC900 shows four peaks at 726.9 eV, 723.8 eV, 716.5 eV, and 710.9 eV (Figure 3g), corresponding to the Fe^3+^ 2p_1/2_, Fe^2+^ 2p_1/2_, Fe^3+^ 2p_3/2_, and Fe^2+^ 2p_3/2_ in FeS [33]. In addition, two new peaks of Fe^0^ are depicted at 721.6 and 707.2 eV in FeS-SNC900 [34], which is due to the introduction of excessive Fe species. As revealed in Figure 3f,g, the binding energy of Fe^2+^ 2p_3/2_ and Fe^2+^ 2p_1/2_ in Fe/ZnS-SNC900 is higher than that in FeS-SNC900, which means more charge is removed from Fe sites. Inversely, the binding energy of Zn^2+^ 2p_3/2_ and Zn^2+^ 2p_1/2_ in Fe/ZnS-SNC900 is lower than that in ZnS-SNC900, confirming the electron transfer from FeS to adjacent ZnS components in Fe/ZnS-SNC900 [35,36]. The interaction at heterointerfaces would lead to the optimized adsorption energy of reactants, thereby greatly improving ORR activity.

### 3.2. Electrocatalytic ORR Performance

**Figure 4:**
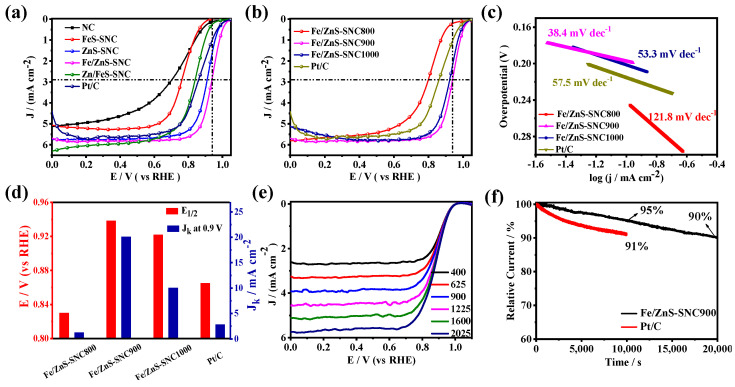
ORR performance in 0.1 M KOH electrolyte. Polarization curves of different samples (**a**) and Fe/ZnS-SNCX (**b**). Tafel slopes (**c**) and histograms of E_1/2_ and J_k_ at 0.9 V vs. RHE (**d**). LSV curves of Fe/ZnS-SNC900 at different rotation rates (**e**). I−t chronoamperometry responses of Fe/ZnS-SNC900 and Pt/C (**f**).

The electrocatalytic performance of the prepared catalysts toward ORR was first evaluated in 0.1 M KOH electrolytes. For comparison, the performance of Pt/C was measured in the same condition. As shown in Figure S4, a well-defined reduction peak at 0.93 V vs. RHE can be observed through the CV curve of Fe/ZnS-SNC900 in O_2_-saturated electrolytes, manifesting its effective ORR catalytic activity. Subsequently, the linear sweep voltammetry (LSV) curves (Figure 4a) exhibit superior ORR activity on Fe/ZnS-SNC, featuring the highest half-wave potential (E_1/2_ = 0.94 V) and diffusion-limited current density (J_L_ = 5.83 mA cm^−2^) among all as-synthesized catalysts. In this case, Fe/ZnS-SNC900 displays the optimal catalytic performance of ORR under the different sulfuration temperatures (Figure 4b). Moreover, it is seen that in Figure 4c, Fe/ZnS-SNC900 shows the lowest Tafel slope of 38.4 mV dec^−1^, which is much lower than those of Fe/ZnS-SNC800 (121.8 mV dec^−1^), Fe/ZnS-SNC1000 (53.3 mV dec^−1^), and Pt/C (57.5 mV dec^−1^), indicating the faster reaction kinetics on Fe/ZnS-SNC900 [37,38]. These results were also confirmed by the higher kinetic current density of 20.1 mA cm^−2^@0.90 V on Fe/ZnS-SNC900 compared to those of Fe/ZnS-SNC800 (1.27 mA cm^−2^), Fe/ZnS-SNC1000 (10.07 mA cm^−2^), and Pt/C catalysts (2.85 mA cm^−2^) (Figure 4d). The ORR polarization curves measured using a rotating disk electrode (RDE) at 400–2025 rpm are shown in Figure 4e, confirming the first-order ORR kinetics on Fe/ZnS-SNC900 [39]. The electron transferred number (n) obtained from the fitted Koutecky–Levitch (K-L) plots was 3.9–4.1 at the potentials of 0.1–0.6 V (Figure S5), demonstrating a 4e^−^ reaction pathway from O_2_ to H_2_O [40]. Next, the electrochemically active surface areas (ECSAs) of different catalysts were determined from the double-layer capacitance (C_dl_) recorded on the non-Faraday region (Figure S6). As depicted in Figure S7, the C_dl_ of Fe/ZnS@SNC900 is 24.43 mF cm^−2^, which is higher than those of Fe/ZnS@SNC800 (3.05 mF cm^−2^), Fe/ZnS@SNC1000 (19.18 mF cm^−2^), and Pt/C (10.13 mF cm^−2^), indicating the significantly improved ECSA of Fe/ZnS@SNC900 (610.75 cm^2^) compared with Fe/ZnS@SNC800 (76.25 cm^2^), Fe/ZnS@SNC1000 (479.5 cm^2^), and Pt/C (253.25 cm^2^) catalysts due to the strong synergistic effects between the FeS and ZnS species. The i-t curves in Figure 4f show that Fe/ZnS-SNC900 exhibits better operating stability, retaining a relative current density of 95% after 10,000 s, which is better than that of Pt/C (91%). The comparisons of analogous non-noble metal catalysts are summarized in Table S1, further confirming the superb ORR performance of Fe/ZnS-SNC900 compared to others reported under alkaline conditions.

**Figure 5:**
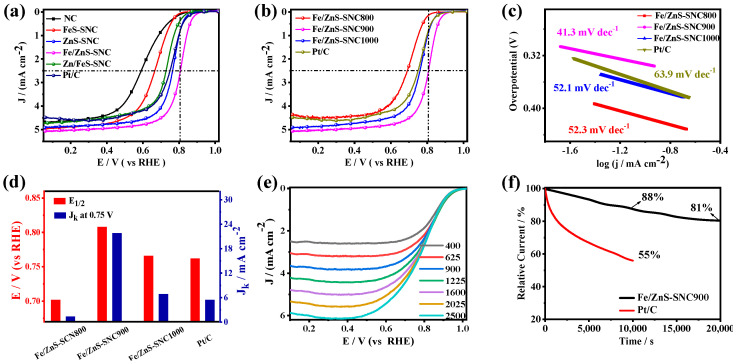
ORR performance in 0.5 M H_2_SO_4_ electrolytes. Polarization curves of different samples (**a**) and Fe/ZnS-SNCX (**b**). Tafel slopes (**c**) and histograms of E_1/2_ and J_k_ at 0.75 V vs. RHE (**d**). LSV curves of Fe/ZnS-SNC900 at different rotation rates (**e**). I−t chronoamperometry responses of Fe/ZnS-SNC900 and Pt/C (**f**).

The ORR performance of different catalysts was also investigated in 0.5 M H_2_SO_4_ solution. The CV curve of Fe/ZnS-SNC in Figure S8 shows a significant cathodic peak at 0.81 V vs. RHE, which belongs to the reduction of O_2_. The LSV curves in Figure 5a indicate that Fe/ZnS-SNC possesses outstanding ORR activity with an E_1/2_ of 0.81 V, which is higher than those of NC (E_1/2_ = 0.60 V), FeS-SNC (E_1/2_ = 0.67 V), ZnS-SNC (E_1/2_ = 0.77 V), Zn/FeS-SNC (E_1/2_ = 0.73 V), and benchmark Pt/C catalysts (E_1/2_ = 0.76 V). Moreover, the Fe/ZnS-SNC900 shows an optimal ORR activity among the compared catalysts with different sulfuration temperatures (Figure 5b). The ORR kinetics of the prepared catalysts was evaluated using Tafel slope and J_k_ values. As observed in Figure 5c, the Tafel slope decreases sequentially in the following order: Pt/C (63.9 mV dec^−1^) > Fe/ZnS-SNC1000 (52.1 mV dec^−1^) > Fe/ZnS-SNC800 (52.3 mV dec^−1^) > Fe/ZnS-SNC900 (39.3 mV dec^−1^), which is consistent with the LSV results and validates the faster ORR kinetics on Fe/ZnS-SNC900. Furthermore, the Fe/ZnS-SNC900 presents a kinetic current density (J_k_) of 21.8 mA cm^−2^, which is superior to that of the Pt/C (Figure 5d, 5.48 mA cm^−2^) at 0.75 V vs. RHE. Additionally, the LSV curves of Fe/ZnS-SNC900 at different speeds (400–2500 rpm) are depicted in Figure 5e. The electron transferred numbers (n) calculated from the fitted K-L plots are determined to be 3.9–4.1 at the potential of 0.1–0.5 V (Figure S9), revealing a near-4e^-^ ORR pathway for O_2_ reduction to H_2_O in acidic conditions [41]. The stability of Fe/ZnS-SNC900 and Pt/C were tested via chronoamperometry in different acidic electrolytes (Figure 5f). It can be shown that the Fe/ZnS-SNC900 delivers a current retention of 81% after 20,000 s operation in 0.5 M H_2_SO_4_ solution, which was considerably better than that of Pt/C (55%) after 10,000 s. The excellent ORR activity and durability of Fe/ZnS-SNC900 in acid conditions demonstrate its potential application prospects.

### 3.3. Zn-Air Batteries and PEMFC

**Figure 6:**
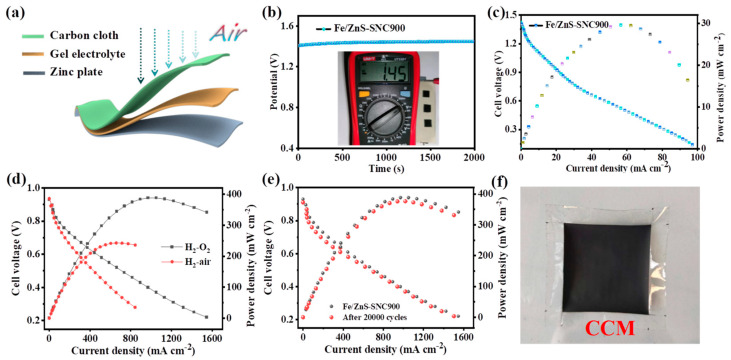
Zn-air battery tests (**a**–**c**). Schematic diagram of the assembled flexible ZABs (**a**). Open-circuit voltage curve of ZAB with Fe/ZnS-SNC900 as air cathodes (**b**). The discharge polarization and power density curves of Fe/ZnS-SNC900-equipped flexible ZAB (**c**). Fuel cell tests (**d**–**f**). The discharge polarization and the corresponding power density curves of fuel cell using Fe/ZnS-SNC900 as a cathode in H_2_-O_2_ and H_2_-air condition (**d**). Polarization curves of Fe/ZnS-SNC900 as the cathode before and after 20000 cycles ADT (**e**). The photograph of membrane electrode with Pt/C (20 wt.%) and Fe/ZnS-SNC900 as anode and cathode catalysts, respectively (**f**).

In order to demonstrate the practical application, flexible Zn-air batteries (ZABs) were assembled to evaluate the feasibility of carbon cloth-loaded Fe/ZnS-SNC900 as an air electrode (Figure 6a). The open-circuit voltage of ZAB equipped with Fe/ZnS-SNC900 electrodes is found to be 1.45 V (Figure 6b), which is in accordance with the actual measurement value of the voltmeter. The peak power density of Fe/ZnS-SNC900-based flexible ZAB could reach 30.2 mW cm^−2^ (Figure 6c), which is comparable to the previously reported analogous catalysts (Table S2). Two tandem ZABs employing Fe/ZnS-SNC900 as air cathodes can power the red light-emitting diodes (Figure S10), manifesting their great applicability in driving electronic devices. A membrane electrode assembly (MEA) with an active area of 4 cm^2^ was fabricated to evaluate its performance in H_2_-O_2_/air fuel cells. The polarization curves of Fe/ZnS-SNC900 deliver a peak power density of 388.3 and 242.8 mW cm^−2^ at the H_2_-O_2_/air condition, respectively, under the backpressure of 2.0 bar (Figure 6d). The cells assembled with Fe/ZnS-SNC900 cathode were further subjected to accelerated durability tests (ADT) at 0.6–0.9 V. The peak power density of Fe/ZnS-SNC900 declined by about 3.1% after 20,000 cycles, indicating its good stability in practical device applications (Figure 6e). The photograph of a practical membrane electrode with Pt/C (20 wt.%) and Fe/ZnS-SNC900 as anode and cathode catalysts is illustrated in Figure 6f.

## 4. Conclusions

In this work, we proposed a facile method to fabricate an Fe-NH_2_-BDC@ZIF-8 nanocomposite that could be further converted into FeS and ZnS heterojunctions embedded in a N,S-codoped porous carbon electrocatalyst (Fe/ZnS-SNC) via sulfonating strategy. The as-prepared Fe/ZnS-SNC900 exhibits higher catalytic activities for ORR in both alkaline and acidic conditions, which is comparable to the commercial Pt/C and even higher than that of the analogous non-precious metal catalysts ever reported. Moreover, the Zn-air battery and PEMFC using Fe/ZnS-SNC as cathodes demonstrate good discharge performance, showing great application prospects in energy conversion devices. XPS analysis further confirms the electron transfer at the FeS/ZnS heterointerfaces and the resulting boost in ORR catalytic activity from modulating the adsorption energy of intermediates on sulfide sites. This work offers a new avenue for the design of highly efficient heterostructured electrocatalysts for sustainable energy conversion and storage systems.

## Data Availability

Not applicable.

## Supplementary Materials

The following supporting information can be downloaded at: https://www.mdpi.com/article/10.3390/nano13192682/s1, Figure S1: SEM image of ZIF-8; Figure S2: SEM image and element mapping of carbon, oxygen, sulfur, zinc, and iron of Zn/FeS-SNC; Figure S3: The survey XPS spectrum of Fe/ZnS-SNC900; Figure S4: CV curves of Fe/ZnS-SNC900 in O_2_- or N_2_-saturated 0.1 M KOH electrolyte; Figure S5: K-L plots of Fe/ZnS-SNC900 in 0.1 M KOH solution; Figure S6: CV curves at various scan rates of Fe/ZnS-SNC800 (a), 900 (b), 1000 (c), and Pt/C (d) catalysts in the range of 1.02–1.12 V in 0.1 M KOH electrolyte; Figure S7: Double-layer capacitance C_dl_ of Fe/ZnS-SNC800, 900, 1000, and Pt/C catalysts; Figure S8: CV curves of Fe/ZnS-SNC900 in O_2_- or N_2_-saturated 0.5 M H_2_SO_4_ electrolyte; Figure S9: K-L plots of Fe/ZnS-SNC900 in 0.5 M H_2_SO_4_ solution; Figure S10: Photograph of a red light-emitting diode powered by two integrated sandwich-type ZABs in series; Table S1: Comparison of ORR activities in alkaline electrolyte among Fe/ZnS-SNC and other non-noble metal electrocatalysts reported in the literature; Table S2: Comparison of the flexible ZAB performances using Fe/ZnS-SNC and other recently reported ORR electrocatalysts.
