# Supplementary material for: Constructing FeS and ZnS Heterojunction on N,S-Codoped Carbon as Robust Electrocatalyst toward Oxygen Reduction Reaction
Source: Nanomaterials (Basel). 2023 Sep 30;13(19):2682. doi: 10.3390/nano13192682 (PMC10574382; doi:10.3390/nano13192682)
Supplement: Supplementary file 1 [file nanomaterials-13-02682-s001.zip › nanomaterials-2625749-supplementary.pdf]

nanomaterials

Article

# Constructing FeS and ZnS Heterojunction on N, S-Codoped Carbon as Robust Electrocatalyst toward Oxygen Reduction Reaction

**Fenglai Pei [1,†], Min Li [2,†], Yifan Huang [2], Qiuyun Guo [3], Kunming Song [3], Fantao Kong [2,*] and Xiangzhi Cui [2,3,*]**

[1] Shanghai Motor Vehicle Inspection Certification & Tech Innovation Center Co., Ltd., Jiading District, Shanghai 201805, China; fenglaip@smvic.com.cn
[2] Shanghai Institute of Ceramics, Chinese Academy of Sciences, Shanghai 200050, China; limin232@mails.ucas.ac.cn (M.L.); huangyifan231@mails.ucas.ac.cn (Y.H.)
[3] School of Chemistry and Materials Science, Hangzhou Institute for Advanced Study, University of Chinese Academy of Sciences, Hangzhou 310024, China; guoqiuyun22@mails.ucas.ac.cn (Q.G.); songkunming22@mails.ucas.ac.cn (K.S.)
* Correspondence: kongfantao@mail.sic.ac.cn (F.K.); cuixz@mail.sic.ac.cn (X.C.)
† These authors contributed equally to this work.

## Electrochemical measurements

The ORR performance was evaluated on the standard three-electrode system connected with CHI 760E electrochemical workstation (CH Instruments, China) at room temperature. The glassy carbon electrode (GCE, 3 mm in diameter) coated with catalyst was used as working electrode. The ink of catalysts was prepared by dispersing 10 mg of the as-synthesized samples into 1 mL of isopropanol. Then, 30 μL of 5 wt.% Nafion D-520 dispersion was added to the mixture and ultrasound for about 30 minutes. Subsequently, 15 uL of the catalyst ink was dropped onto the surface of the glassy carbon electrode and then dried at room temperature before tests. The ink of Pt/C (20 wt.%) was also prepared by the same method. The loading of the as-prepared catalysts and Pt/C are 0.40 mg cm$^{-2}$ and 0.60 mg cm$^{-2}$ in alkaline and acidic electrolytes, respectively. The Ag/AgCl and carbon rod were used as referenc electrode and counter electrodes, respectively. The Nernst equation ($E_{\mathrm{RHE}} = E_{\mathrm{Ag/AgCl}} + 0.059 \times pH + 0.1989\ \mathrm{V}$) was used to standardized all test potentials to reversible hydrogen electrode (vs. RHE).

The electrocatalytic performance of catalysts was evaluated in 0.1 M KOH or 0.5 M $H_2SO_4$ electrolytes. Linear scanning voltammetry (LSV) curves were recorded by rotating disc electrode (RDE) techniques at a constant rotating speed of 1600 rpm in different electrolytes with the scan rate of 5 mV·s$^{-1}$. The electron transfer number ($n$) of ORR was calculated based on the Koutecky-Levich (K-L) equation:

$$\frac{1}{j} = \frac{1}{j_k} + \frac{1}{j_L} = \frac{1}{j_k} + \frac{1}{B\omega^{1/2}}$$

$$B = 0.62nFD_{O_2}{}^{2/3}\nu^{-1/6}C_0$$

$$j_K = \frac{j_L \times j}{j_L - j}$$

where $j$ is the measured disk current density, $j_L$ and $j_k$ are the diffusion-limiting current densities and kinetic current densities, respectively. $\omega$ is the electrode rotation rate (rad s$^{-1}$). $n$ is the number of electrons transferred per $O_2$ molecule. $C_0$ is the oxygen concentration of electrolyte (1.2 × 10$^{-6}$ mol cm$^{-3}$), $D_{O_2}$ is the diffusion coefficient of $O_2$ (1.9 × 10$^{-5}$ cm$^2$·s$^{-1}$), $v$ is the kinetic viscosity of the electrolyte, and F is Faraday constant (96485 C mol$^{-1}$).

Next, the linear part of the Tafel curves was matched to the Tafel equation ($\eta = a + b\log|j|$), and then the Tafel slope ($b$) was obtained to evaluate the kinetic performance of

the as-prepared catalysts. The long-time stability was evaluated by chronoamperometry in alkaline and acidic media, respectively, with the electrode rotating speed of 1600 rpm.

Cyclic voltammetry (CV) tests were measured in $N_2$/$O_2$-saturated electrolytes with a sweep rate of 10 mV $s^{-1}$. The electrochemically active surface area (ECSA) of the different catalysts was estimated from the electrochemical double layer capacitance ($C_{dl}$) obtained from CV curves in a non-faradaic potential range (1.02-1.12 vs. RHE) at different scan rates (10-30 mV $s^{-1}$). The ECSA can be calculated by the following equation:

$$ECSA = C_{dl}/C_S$$

Where the value of $C_s$ is usually 0.04 mF $cm^{-2}$ in alkaline electrolyte.

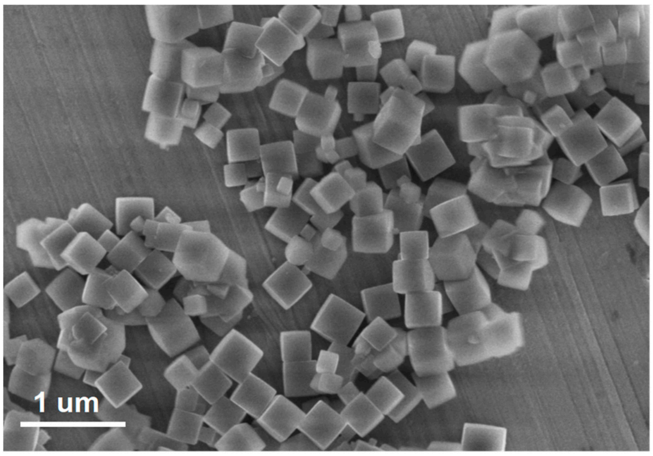


**Figure S1.** SEM image of ZIF-8.

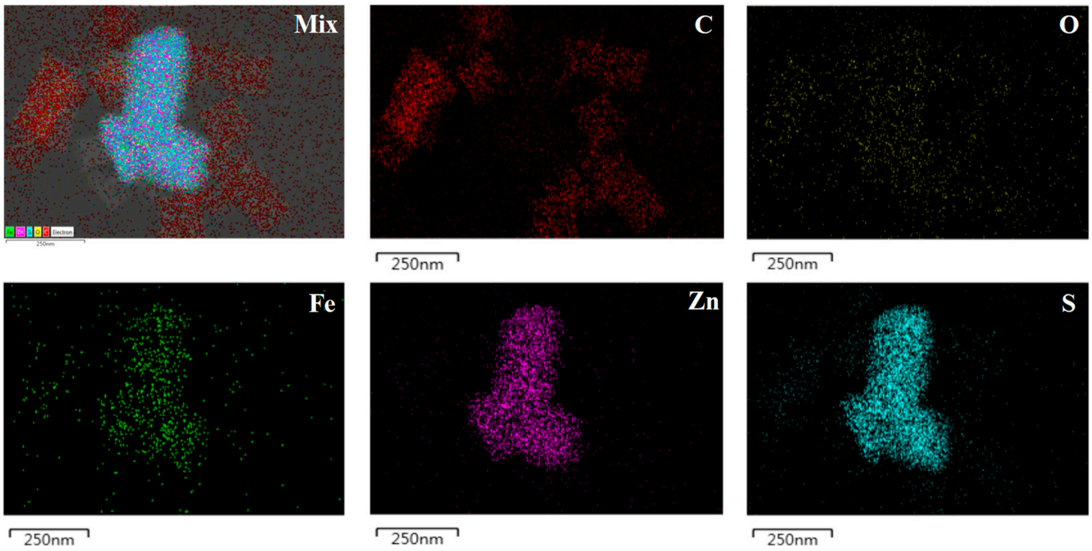


**Figure S2.** SEM image and element mapping of carbon, oxygen, sulfur, zinc and iron of Zn/FeS-SNC.

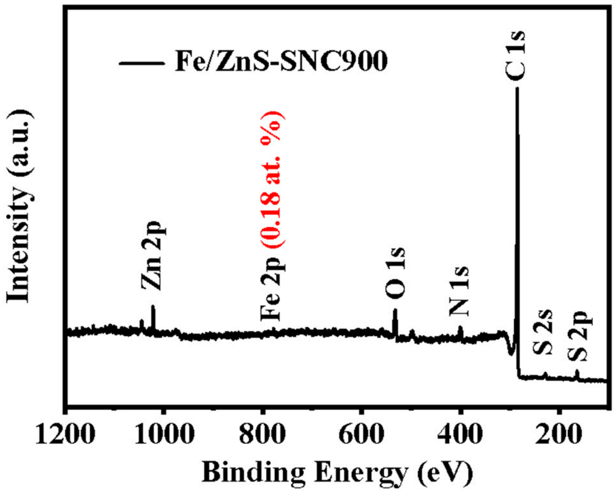


**Figure S3.** The survey XPS spectrum of Fe/ZnS-SNC900.

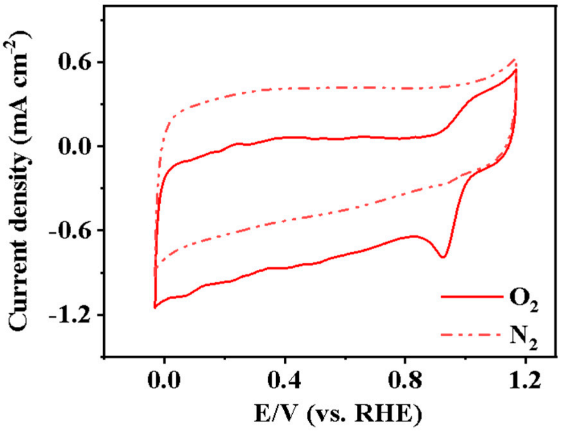


**Figure S4.** CV curves of Fe/ZnS-SNC900 in $O_2$- or $N_2$-saturated 0.1 M KOH solution.

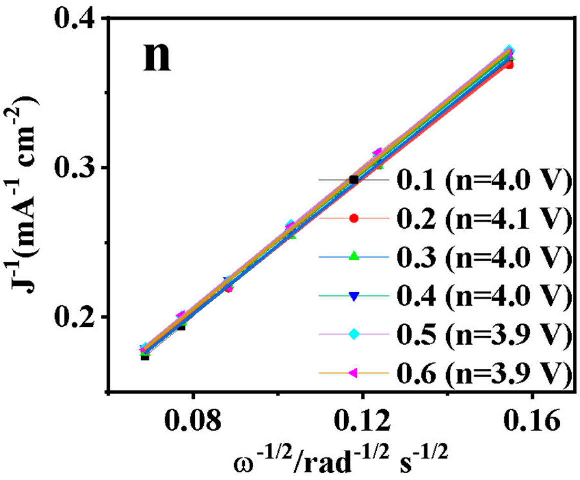


**Figure S5.** K-L plots of Fe/ZnS-SNC900 in 0.1 M KOH solution.

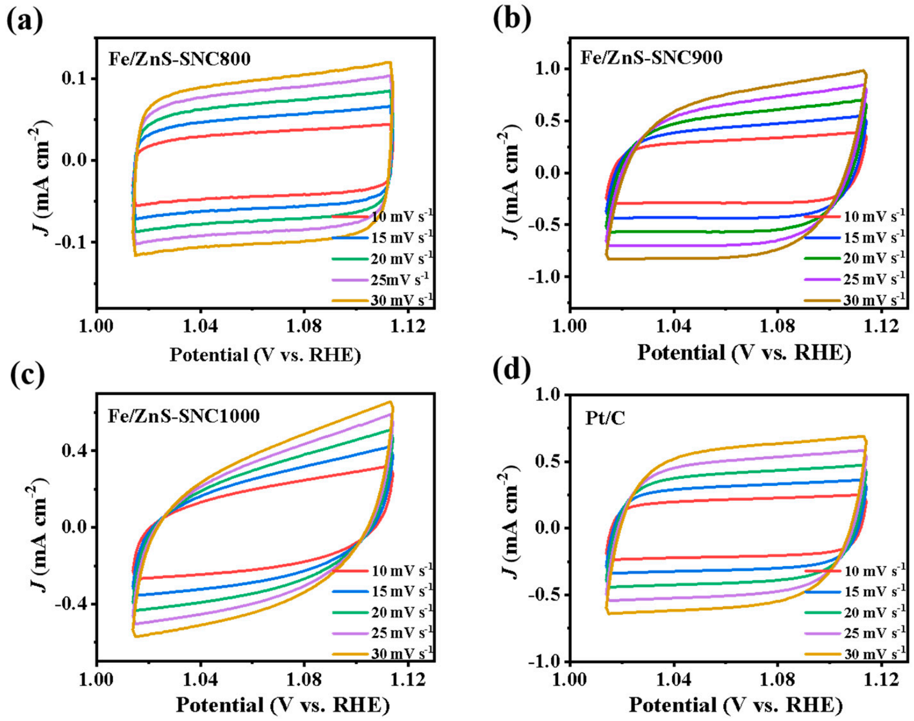


**Figure S6.** CV curves at various scan rates of Fe/ZnS-SNC800 (**a**), 900 (**b**), 1000 (**c**) and Pt/C (**d**) catalysts in the range of 1.02-1.12 V in 0.1 M KOH solution.

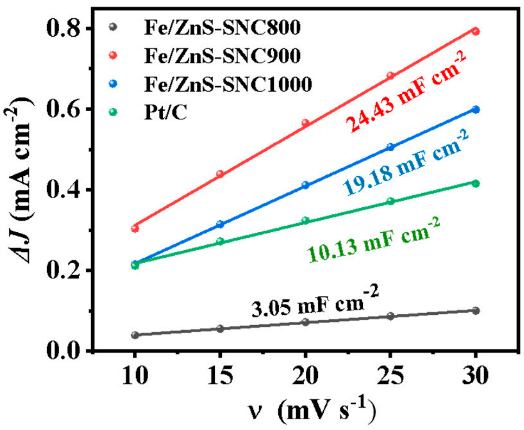


**Figure S7.** Double-layer capacitance $C_{dl}$ of Fe/ZnS-SNC800, 900, 1000 and Pt/C catalysts.

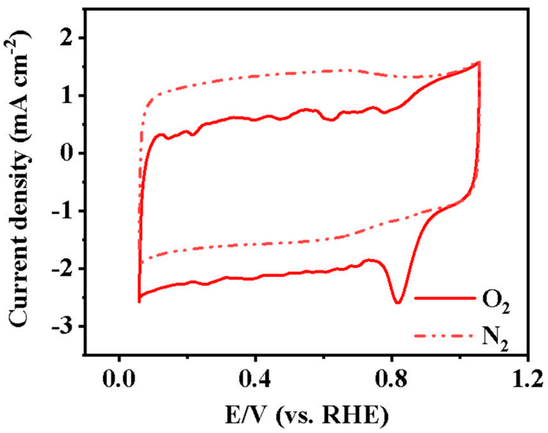


**Figure S8.** CV curves of Fe/ZnS-SNC900 in $O_2$- or $N_2$-saturated 0.5 M $H_2SO_4$ solution.

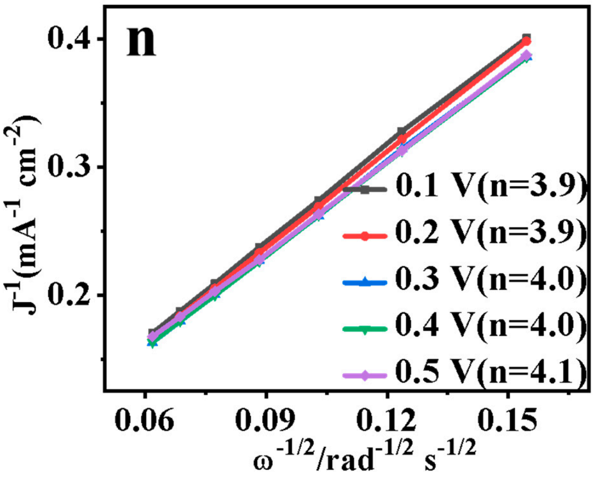


**Figure S9.** K-L plots of Fe/ZnS-SNC900 in 0.5 M $H_2SO_4$ solution.

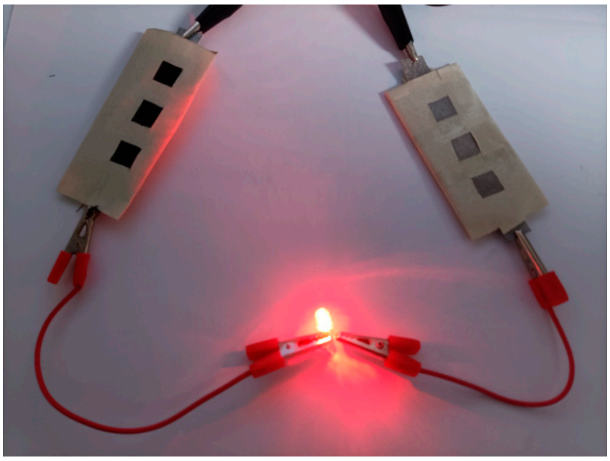

**Figure S10.** Photograph of a red light-emitting diode powered by two integrated sandwich-type ZABs in series.

**Table S1.** Comparison of ORR activities in alkaline electrolyte among Fe/ZnS-SNC and other non-noble metal electrocatalysts reported in the literature.

| Catalysts | Electrolyte | $E_{onset}$ (V) | $E_{1/2}$ (V) | References |
|---|---|---|---|---|
| $FeN_4$-Ten | 0.1 M KOH | 0.922 | 0.867 | *Adv. Mater., 2022, 3, 2202714* |
| (Zn, Cu)−NC | 0.1 M KOH | ~ | 0.880 | *Adv. Funct. Mater., 2022, 32, 2203471* |
| PtFeNC | 0.1 M KOH | 1.050 | 0.895 | *Appl. Catal. B: Environ.* 2021, 286, 119891 |
| Fe,Mn/N-C | 0.1 M KOH | 0.979 | 0.928 | *Nat. Commun.* 2021, 12, 1734 |
| Ru-SAS/SNC | 0.1 M KOH | 0.998 | 0.861 | *J. Am. Chem. Soc. 2022, 144, 2197-2207* |
| Fe/Ni-N-C | 0.1 M KOH | 1.005 | 0.861 | *Appl. Catal. B: Environ.* 2021, 285, 119778 |
| Fe-N/P-C-700 | 0.1 M KOH | 0.941 | 0.867 | *J. Am. Chem. Soc.* 2020, 142, 2404 |
| Fe, Co SAs-PNCF | 0.1 M KOH | 1.040 | 0.930 | *Nano Energy, 2022, 93, 106793* |
| COF@MOF800 | 0.1 M KOH | 0.830 | 0.730 | *Small Structures, 2022, 3, 2100225* |
| $FeN_4$-O-NCR | 0.1 M KOH | 1.05 | 0.940 | Energy Environ. Sci., 2022, 15, 2619-2628 |
| Fe-SA/Meso-C | 0.1 M KOH | ~ | 0.926 | *Carbon, 2022, 191, 393-402* |
| ZFP-800 | 0.1 M KOH | 1.028 | 0.875 | *J. Colloid Interf. Sci. 2022, 608, 446-458* |
| **Fe/ZnS-SNC** | **0.1 M KOH** | **1.080** | **0.938** | **This work** |

**Table S2.** Comparison of the flexible ZAB performances using Fe/ZnS-SNC and other recently reported ORR electrocatalysts.

| Catalyst | Open circuit voltage (V) | Peak power density ($mW\ cm^{-2}$) | References |
|---|---|---|---|
| $FeP/Fe_2O_3$ @NPCA | 1.42 | 40.8 | *Adv. Mater.* 2020, 32, 2002292 |
| SAFe-SWCNT | 1.36 | 33 | *Appl. Catal. B: Environ.* 2021, 294, 120239 |
| ODAC-CoO-30 | 1.41 | 42 | *Adv. Funct. Mater.* 2021, 31, 2101239 |
| FeCo/Se-CNT | 1.41 | 37.5 | *Nano Lett.* 2021, 21, 2255 |
| SAFe-SWCNT | 1.36 | 33 | *Appl. Catal. B: Environ.* 2021, 294, 120239 |
| $FeNi_3$@NC | 1.38 | – | *Appl. Catal. B: Environ.* 2020, 268, 118729 |
| ODAC-CoO-30 | 1.41 | 42 | *Adv. Funct. Mater.* 2021, 31, 2101239 |
| **Fe/ZnS-SNC** | **1.45** | **30.8** | **This work** |
